# Note on the Preparation of Sodium Amalgam in the Form of Pellets

**DOI:** 10.6028/jres.064A.012

**Published:** 1960-02-01

**Authors:** H. S. Isbell, H. L. Frush, N. B. Holt

## Abstract

A procedure is described for the preparation of sodium amalgam in the form of pellets.

In connection with the development of methods for the synthesis of radioactive carbohydrates,^1^ a procedure and apparatus were previously described [1, 2, 3]^2^ for the reduction of semimicro quantities of aldonic lactones to sugars. The method employs sodium amalgam together with a slightly soluble, acid salt (sodium binoxalate) as buffer.^3^ The amalgam is used in the form of pellets made by dropping the molten amalgam into a “shot tower” of oil. Because other workers have had difficulty in making these pellets, the procedure is now given in detail.

The amalgam is prepared in a 500-ml, round-bottomed, stainless-steel flask having a single neck, with a 24/40 standard-taper joint (outer),^4^ and a thermometer inlet. The joint is fitted with a stainless-steel stopper, which has an inlet tube bent at right angles to the stopper and covered with asbestos for convenience in handling. The flask is held in a sturdy, asbestos-covered clamp, which serves as a handle for the vessel during the heating step. An alundum (Soxhlet extraction) thimble 45 mm in diam, in the bottom of which six 1.5-mm holes have been drilled, is held in a second asbestos-covered clamp. The oil bath is a thick-walled heat-resistant glass jar, 6 in. in diam and 18 in. high, containing paraffin oil to within 3 in. of the top (see fig. 1).

To prepare the amalgam, a weighed amount of mercury is placed in the flask, into which a continuous stream of dry nitrogen is passed by means of the inlet tube of the stopper. The required amount of sodium^5^ is weighed under paraffin oil, and then cut into pieces just small enough to be readily slipped through the neck of the flask. Each piece is rinsed in a hydrocarbon solvent, such as heptane or toluene, quickly blotted dry, and dropped through the neck of the flask into the mercury; the stopper is immediately replaced. The sodium reacts quickly with the mercury and may be added fairly rapidly because of the atomosphere of nitrogen. After the addition of the sodium is completed, the flask is heated with a Meeker burner until the amalgam is entirely molten. (The presence of remaining solid particles in the amalgam maybe detected by the sound of their impact on the walls of the flask when it is given a gentle, swirling motion.) While the amalgam is being prepared, the alundum thimble is heated over another Meeker burner by a second operator. The hot thimble is then so clamped that its bottom is 1 to 2 in. above the surface of the oil, and the molten amalgam is poured into the thimble from the thermometer inlet of the flask. The amalgam flows through the holes in the bottom of the thimble, drops through the oil, and collects at the bottom of the oil bath as small, rather flat pellets.

Optimal conditions for the production of smooth pellets must be determined by trial. If the flask and thimble have not been sufficiently heated, the amalgam may solidify in the thimble. If the holes in the thimble are too large, the product may be somewhat “thready.” However, once the optimal conditions have been established, the procedure may be repeated without difficulty.

The entire operation must be performed in an efficient hood. The amalgam is stored under paraffin oil in a wide-mouthed, screw-capped bottle (see fig. 2). Pellets are removed as needed, weighed under oil, and rinsed with an inert, volatile solvent immediately before use.

## Figures and Tables

**Figure 1:**
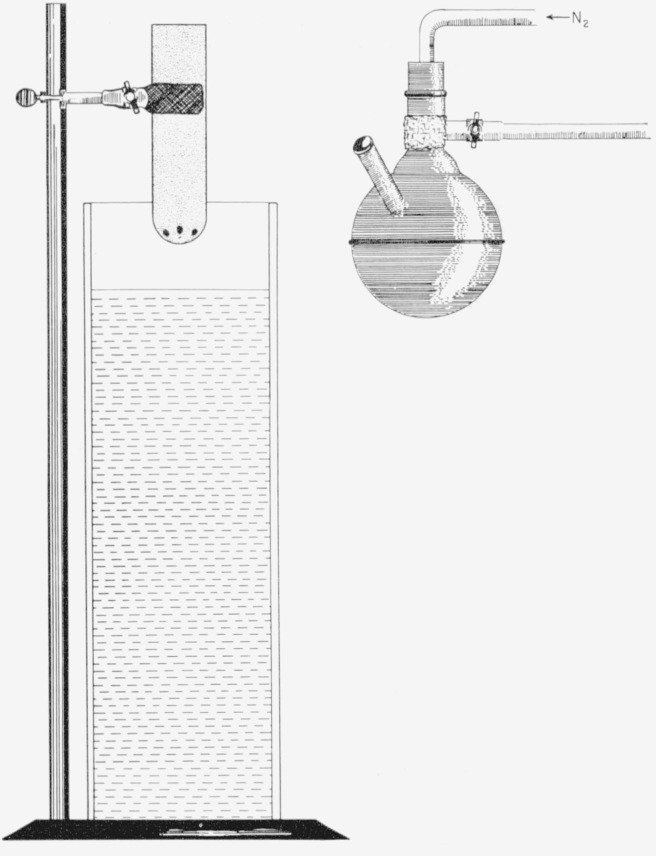
Apparatus used in the preparation of sodium amalgam pellets.

**Figure 2:**
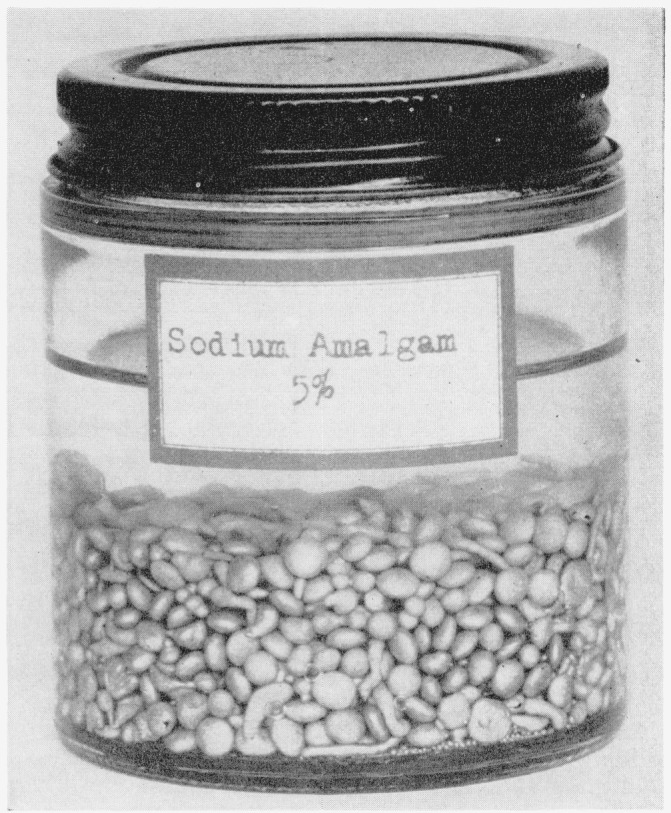
Sodium amalgam pellets.

## Footnotes

1 A project sponsored by the Division of Research of the Atomic Energy Commission.

2 Figures in brackets indicate the literature references at the end of this paper.

3 The reduction procedure has also been modified for use with macro quantities of lactones [4].

4 Scientific Glass Company, Bloomfield, N.J.

5 For the reduction of aldonic lactones under the conditions employed in this laboratory a 5-percent amalgam has been found to be satisfactory.
